# Linolenic Acid Plus Ethanol Exacerbates Cell Death in *Saccharomyces cerevisiae* by Promoting Lipid Peroxidation, Cardiolipin Loss, and Necrosis

**DOI:** 10.3390/life12071052

**Published:** 2022-07-14

**Authors:** Berenice Eridani Olmos-Orizaba, José Santos Arroyo-Peñaloza, Lorena Martínez-Alcántar, Rocío Montoya-Pérez, Alberto Flores-García, Alain Raimundo Rodríguez-Orozco, Elizabeth Calderón-Cortés, Alfredo Saavedra-Molina, Jesús Campos-García, Christian Cortés-Rojo

**Affiliations:** 1 Instituto de Investigaciones Químico-Biológicas, Universidad Michoacana de San Nicolás de Hidalgo, Morelia 58030, Mexico; 1028309h@umich.mx (B.E.O.-O.); 1230382c@umich.mx (J.S.A.-P.); lorena.martinez@umich.mx (L.M.-A.); rocio.montoya@umich.mx (R.M.-P.); alberto.flores@umich.mx (A.F.-G.); francisco.saavedra@umich.mx (A.S.-M.); jesus.campos@umich.mx (J.C.-G.); 2 Facultad de Ciencias Médicas y Biológicas “Dr. Ignacio Chávez”, Universidad Michoacana de San Nicolás de Hidalgo, Morelia 58020, Mexico; alain.rodriguez@umich.mx; 3 Facultad de Enfermería, Universidad Michoacana de San Nicolás de Hidalgo, Morelia 58000, Mexico; elizabeth.calderon@umich.mx

**Keywords:** PUFA, yeast, alcohol, apoptosis, necrosis, mitochondria, oxidative stress, linolenic acid

## Abstract

Polyunsaturated fatty acids (PUFA) hypersensitize yeast to oxidative stress. Ethanol accumulation during fermentation is another factor that induces oxidative stress via mitochondrial dysfunction and ROS overproduction. Since this microorganism has raised growing interest as a PUFA factory, we have studied if the combination of PUFA plus ethanol enhances yeast death. Respiration, ROS generation, lipid peroxidation, mitochondrial cardiolipin content, and cell death were assessed in yeast grown in the presence of 10% ethanol (ETOH) or linolenic acid (C18:3), or ethanol plus C18:3 (ETOH+C18:3). Lipid peroxidation and cardiolipin loss were several-fold higher in cells with ETOH+C18:3 than with C18:3. On the contrary, ETOH tended to increase cardiolipin content without inducing changes in lipid peroxidation. This was consistent with a remarkable diminution of cell growth and an exacerbated propidium iodide staining in cells with only ETOH+C18:3. The respiration rate decreased with all the treatments to a similar degree, and this was paralleled with similar increments in ROS between all the treatments. These results indicate that PUFA plus ethanol hypersensitize yeast to necrotic cell death by exacerbating membrane damage and mitochondrial cardiolipin loss, independent of mitochondrial dysfunction and ROS generation. The implications of these observations for some biotechnological applications in yeast and its physiology are discussed.

## 1. Introduction

*Saccharomyces cerevisiae* is a Crabtree-positive yeast that produces ethanol to overflow pyruvate excess during glucose oxidation. Once glucose decreases to a certain threshold, *S. cerevisiae* utilizes ethanol as a respiratory substrate [1]. Another feature of *S. cerevisiae* is its membrane fatty acid composition, which consists of only saturated and monounsaturated fatty acids (SFA and MUFA, respectively), and has an absence of polyunsaturated fatty acids (PUFA) [2]. This confers yeast membranes a high resistance to lipid peroxidation since SFA and MUFA are not prone to free radical attack due to the absence of bis-allylic hydrogens [3]. Nevertheless, yeast can incorporate PUFA from its environment into its membranes [4]. PUFA in mitochondrial membranes significantly enhances the sensitivity of yeast mitochondria to lipid peroxidation, leading to the impairment of electron transport chain (ETC) function. In contrast, yeast with native fatty acid composition is highly resistant to the effects of oxidative stress on mitochondria due to an insensitivity to lipid peroxidation [5].

Another challenge for yeast is ethanol accumulation in its surrounding media. Biological membranes are one of the main targets of ethanol. Hence, an adaptive response occurs in the yeast plasma membrane to enhance ethanol tolerance by modifying lipid composition and fluidity. Ethanol increases membrane fluidity, leading to a decrease in membrane integrity [6]. To counteract membrane fluidization, the content of oleic acid (C18:1) increases in the plasma membrane [7], along with the ergosterol/phospholipid ratio [8]. In turn, the increase in ergosterol enhances the activity of proton-pumping ATPase, which establishes a transmembrane proton gradient that drives several vital physiological functions in yeast. It is believed that enhanced ATPase activity participates in ethanol tolerance by counteracting a proton influx elicited by ethanol [9]. Ethanol induces apoptotic cell death by interfering with mitochondrial dynamics [10]. Moreover, ethanol causes oxidative stress and mitochondrial dysfunction by disrupting mitochondrial iron homeostasis and increasing ROS production [11]. On the other hand, cardiolipin is a phospholipid in the inner mitochondrial membrane that is crucial for the function of the ETC, and thus, for cell survival. Cardiolipin is highly susceptible to lipid peroxidation by containing four acyl chains [12]. In yeast, PUFA are readily incorporated into cardiolipin when present in the culture media, and cardiolipin hydroperoxides are produced even in the absence of an oxidative stimulus [13]. This has the potential to impair mitochondrial function since cardiolipin peroxides disrupt the interactions of the ETC with the inner mitochondrial membrane [14].

Yeast is a potential factory for the synthetic production of PUFA via metabolic engineering [15,16]. Increasing the unsaturation of yeast membranes by augmenting the content of oleic acid has been shown to increase ethanol tolerance [17]. However, increased PUFA content in membranes with linoleic (C18:2) or linolenic acid (C18:3) does not enhance ethanol tolerance [18]. In this work, we hypothesize that the presence of PUFA plus ethanol exacerbates cell death in yeast by inducing lipid peroxidation, cardiolipin loss, mitochondrial dysfunction, and necrosis. This is postulated on the basis of ethanol toxicity in yeast via mitochondrial oxidative stress [11], increased susceptibility to oxidative stress in yeast enriched with PUFA [5], and the essentiality of cardiolipin for mitochondrial function [12]. It was found that treatment with C18:3 plus 10% ethanol stimulates necrotic cell death, lipid peroxidation, and cardiolipin loss to a higher degree than treatment with C18:3 alone. On the contrary, ethanol alone tends to increase cardiolipin and does not enhance lipid peroxidation. Moreover, deleterious alterations in respiration and ROS levels were induced to the same extent with any of these treatments. These data suggest that decreased mitochondrial function and ROS overproduction are not factors responsible for the enhanced toxicity of ethanol plus C18:3, but exacerbation in both lipid peroxidation and cardiolipin loss are crucial events that lead to aggravated cell death by necrosis.

## 2. Materials and Methods

### 2.1. Yeast Strains and Growth Conditions

The haploid *S. cerevisiae* strain, BY4741 (*Mat α*; *his3Δ1*; *leu2Δ0*; *met15Δ0*; *ura3Δ0*; WT), was obtained from EUROSCARF (Frankfurt, Germany). The cultures (OD_600nm_ = 0.03) were grown in liquid YPD medium (1% yeast extract, 5% peptone, and 2% dextrose) with orbital shaking at 250 rpm and a temperature of 30 °C. Twelve hours later, 10% (*v*/*v*) ethanol was added. Cells were harvested in the early stationary phase (~20 h). For the manipulation of membrane fatty acid content, 1 mM linolenic acid (C18:3) solubilized with 5% (*v*/*v*) Igepal CA-630 was added to YPD medium right before the inoculation with yeast [5].

### 2.2. Determination of Cell Growth

Cell growth was recorded in the prepared cultures, as described above, by measuring the OD_600nm_ every two hours for up to 24 h in a Shimadzu UV2550 spectrophotometer.

### 2.3. Evaluation of Cell Death

Propidium iodide (PI) (Thermo Fisher Scientific, Waltham, MA, USA) was used to assess the percentage of cells that underwent necrosis. The cells were harvested and resuspended in 200 µL of phosphate buffered saline (PBS), and two 1:10 dilutions were made with a binding buffer. The cells were incubated with 1 µL PI for 15 min in the dark at room temperature. This mixture was centrifuged and resuspended at a final volume of one milliliter. Fluorescence was quantified using an Accuri-C6 flow cytometer (BD Biosciences, San Jose, CA, USA). PI fluorescence was assessed with the FL2 channel at 518/617 nm. Forward vs. side scatter (FSC vs. SSC) gating was done to discard cellular debris with low FSC. Positive controls of cell death were carried out by stressing cells with heat shock (50 °C for 90 min). This resulted in an increase in dead cells from 0.2% to 55.2% in control yeast and from 50.8 to 88.3% in C18:3 + ETOH cells (see Supplementary Figure S1).

### 2.4. Lipid Peroxidation Measurement

Lipid peroxidation levels were evaluated in cells by the thiobarbituric acid (TBA) method [19]. Twenty-five mg cells were mixed with 2 mL of an acid mixture containing 0.25 M HCl, 15% (*w*/*v*) trichloroacetic acid, and 0.375% (*w*/*v*) TBA. This mixture was vortexed for 5 s, boiled for 15 min, cooled to room temperature, and centrifuged at 5000 rpm for 5 min. The absorbance of the supernatant was measured at 532 nm on a Shimadzu UV2550 spectrophotometer. The results were calculated using the molar extinction coefficient of malondialdehyde and expressed in nanomoles of TBA-reactive species (TBARS) per milligram cells.

### 2.5. Mitochondria Isolation

Mitochondria were isolated according to the method by Peña et al. [20], with modifications. Cells were harvested and washed twice with deionized water at 5000 rpm for 5 min. The cell pellet was resuspended in a medium containing 0.6 M mannitol, 5 mM MES (10 mM 2-(N-morpholino)ethanesulfonic acid), and 0.1% bovine serum albumin (pH 6.8 with triethanolamine). The cells were resuspended at a ratio of 0.4 g cells/mL medium. This suspension was mixed with 50% (*v*/*v*) glass beads (0.5 mm diameter) and vortexed at high speed for ten cycles of 1 min vortexing–1 min ice. The cell homogenate was centrifuged at 2590 rpm for 5 min. The pellet was discarded and the supernatant was centrifuged at 12,000 rpm for 10 min. Centrifugations were carried out at 4 °C in a Sorvall RC 6+ centrifuge with the SS-34 rotor. The mitochondrial protein concentration was determined by the Biuret method. The intactness of the mitochondrial preparations was assessed by measuring the rate of respiration in phosphorylating state 3 (stimulated with ADP) and state 4 (corresponding to the rate of idling respiration after ADP exhaustion). In our hands, control mitochondria exhibited respiratory control ratios (state 3/state 4) above 3.0 (see Supplementary Figure S2).

### 2.6. Determination of Cardiolipin Levels

Cardiolipin levels were assessed in isolated mitochondria according to the method by Gallet et al. [21]. Ten mg/mL of mitochondrial protein was incubated with 150 µM 10-N-nonyl acridine orange bromide for 15 min at 20 °C in a buffer containing 0.6 M mannitol and 10 mM tris/HCl, pH 7.4. This mixture was centrifuged at 30,000× *g* for 5 min. The unbounded dye in the supernatant was measured by determining its absorbance at 495 nm. The free dye concentration was calculated from a calibration curve obtained with known 10-N-nonyl acridine orange concentrations (0–10 µM). The concentration of cardiolipin was estimated by subtracting the total amount of dye added to the mitochondrial suspension minus the amount of unbounded dye, taking into consideration a stoichiometry of 2 mols of 10-N-nonyl acridine orange/mol cardiolipin. Positive controls for this method were done consisting of a calibration curve with increased concentrations of cardiolipin liposomes vs. the absorbance of acridine orange at 495 nm. This curve shows an increase in acridine orange absorbance that is proportional to the concentration of cardiolipin liposomes, which was saturable at supraphysiological concentrations above 0.2 mg/mL cardiolipin (Supplementary Figure S3).

### 2.7. Evaluation of Oxygen Consumption

The in situ mitochondrial respiration was evaluated in whole cells. After 20 h of growth, the cells were starved overnight by transferring them to sterile deionized water in order to exhaust endogenous respiratory substrates. Twelve-and-a-half mg of cells were resuspended in MES-TEA buffer (10 mM 2-(N-morpholino)ethanesulfonic acid, pH 6.0 with triethanolamine) and added to a sealed glass chamber with constant stirring. Oxygen consumption determinations started after the addition of 10 mM of glucose (state 4). Two minutes later, 15 μM of carbonyl cyanide m-chlorophenylhydrazone (CCCP) was added to stimulate uncoupled respiration (state U). One µg antimycin was added at the end of the determinations to abolish ETC activity for discrimination between non-mitochondrial and mitochondrial oxygen consumption. Under our experimental conditions, respiration with antimycin A was negligible (Figure 5A), thereby it was assumed that whole oxygen consumption in yeast was due to mitochondrial ETC activity. The oxygen consumption rate was determined at room temperature using a Clark-type oxygen electrode coupled to a YSI 5300 oxygen monitor and a computer for data acquisition. 

### 2.8. Determination of ROS Levels

The cells were washed four times with deionized water and resuspended in 1 mL MES-TEA buffer (pH 6.0). One mg of cells were incubated with constant stirring at 4 °C with 12.5 mM of 2′,7′-dichlorodihydrofluorescein diacetate (H_2_DCFDA), a cell-permeable probe for ROS. After 20 min, the cells were washed with 50 mM of KH_2_PO_4_ buffer (pH 7.6) to remove the ROS probe remaining outside the cells. The cells were placed in a quartz cuvette at a final volume of 2 mL. Basal fluorescence was followed for 1 min (*λ*_ex_ 485 nm; *λ*_em_ 520 nm). Then, ROS production was stimulated by adding 10 mM of dextrose as a substrate, and the changes in the fluorescence were further followed for 15 min. Fluorescence changes were evaluated in a Shimadzu RF-5301PC. The data were calculated by subtracting the final fluorescence obtained after 15 min of dextrose addition minus the fluorescence detected before dextrose addition, and the result was divided by 15. The results were expressed as the change in arbitrary fluorescence units (AFU) per minute per mg of protein. 

### 2.9. Statistical Analysis

Data are expressed as the mean ± standard error of *n* ≥ 5. Statistical significances: * sign denotes statistically significant differences vs. Control; **¶** sign denotes statistically significant differences vs. C18:3; **§** sign denotes statistically significant differences vs. ETOH; **#** sign denotes statistically significant differences vs. C18:3 + ETOH; and n.s. means non-statistical significance (*p* < 0.05, one-way ANOVA, followed by Tukey’s post-hoc test).

## 3. Results

### 3.1. Influence of PUFA on the Effects of Ethanol on Yeast Growth and Cell Death

**Figure 1:**
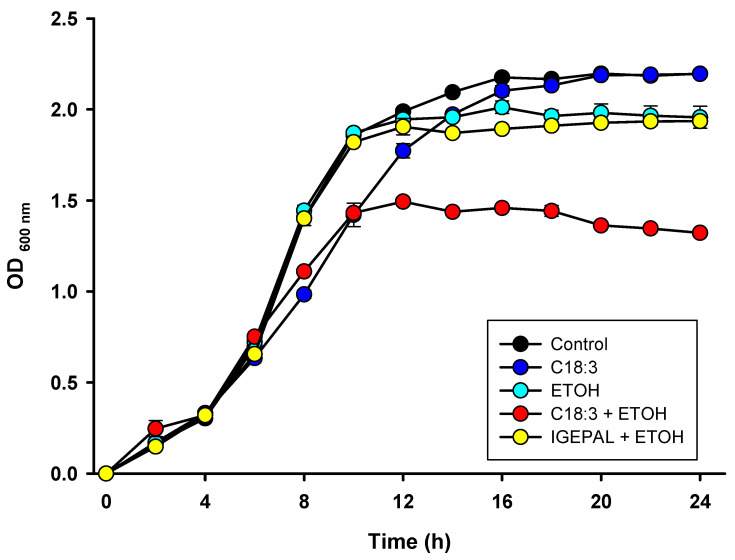
Effects of ethanol and C18:3 on yeast growth. Cell growth was assessed by recording the optical density at 600 nm (OD _(600nm)_) for 24 h cultures every two hours. Ethanol was added at the 12th hour of growth, while C18:3 was added at the beginning of the growth.

Cell growth and death were evaluated to assess whether the presence of PUFA plus ethanol in the culture media exacerbates cell death. Twenty-four-hour growth curves (Figure 1) show that exponential growth in cells with ethanol (ETOH, cyan circles) stops at 12 h of incubation, corresponding to the time at which ethanol was added to the culture. In contrast, the control cells (black circles) keep growing for up to 16 h. There was a slower growth in the cells with linolenic acid (C18:3, blue circles) in comparison to the control group; however, these cells reached the same optical density (OD) as the control cells at 20 h. More deleterious effects were observed in the cells with linolenic acid plus ethanol (C18:3 + ETOH, red circles) since these cells exhibited, as expected, a similar decrease in their growth compared to the C18:3 cells before the addition of ethanol, but ethanol addition at 12 h immediately stopped the growth, thereby yielding the lowest OD among all the treatments. To discard that the deleterious effects of linolenic acid plus ethanol treatment on cell growth were due to the detergent used to solubilize linolenic acid, cells were grown in the presence of the detergent IGEPAL CA-630 and ethanol (IGEPAL + ETOH, yellow circles). IGEPAL + ETOH cells exhibited similar growth as the ETOH cells, indicating that impaired growth by C18:3 + ETOH was not due to the presence of IGEPAL CA-630.

**Figure 2:**
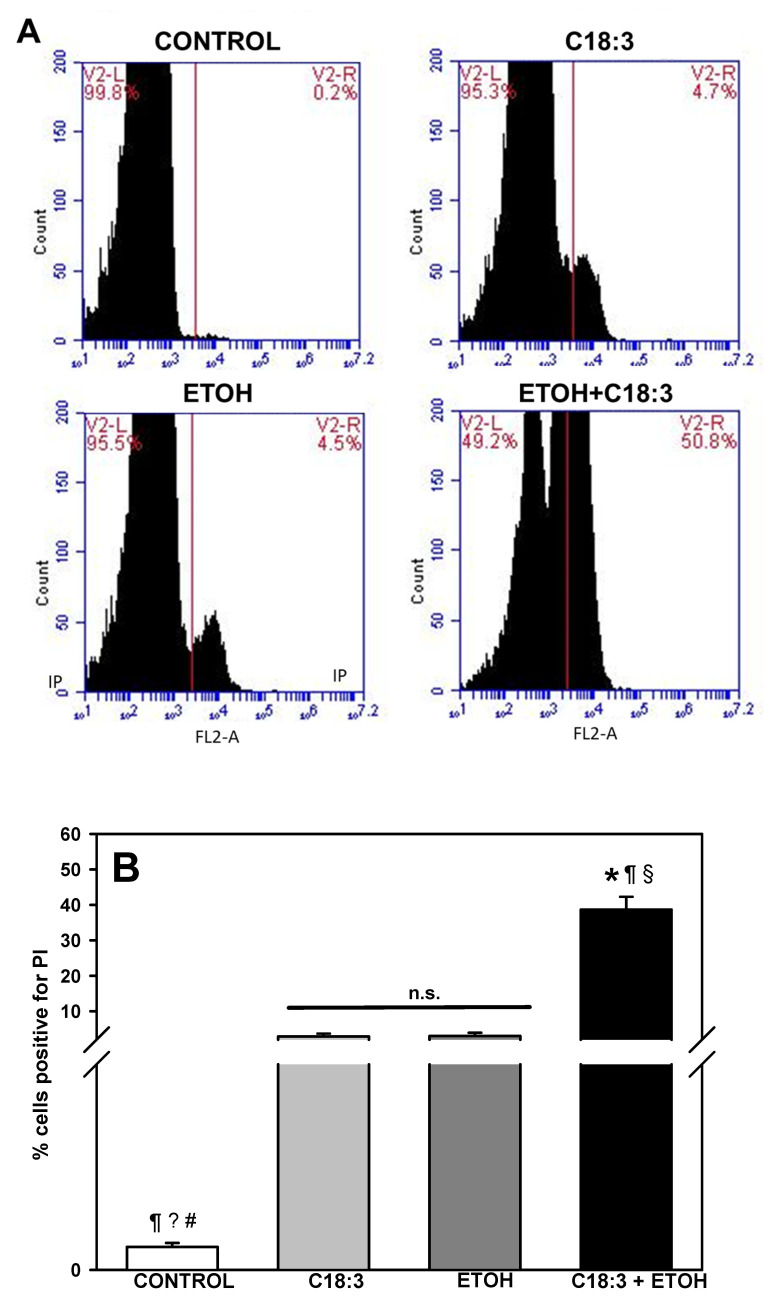
Effects of ethanol and C18:3 on yeast cell death. (**A**) Representative flow cytometry univariate histograms analyzing negative (V2-L region) and positive (V2-R region) populations of PI staining. PI staining was analyzed with the FL2-A (518/617) fluorescence channel. The x axis represents the propidium iodide fluorescence in arbitrary units. (**B**) Quantification of the percentage of cells in the V2-L region. The data are presented as the mean ± s.e. of *n* ≥ 4. * *p* < 0.05 vs. CTRL; ^¶^ *p* < 0.05 vs. C18:3; ^§^ *p* < 0.05 vs. ETOH; ^?,#^ *p* < 0.05; n.s. non-statistical significance (one-way ANOVA with Tukey’s post-hoc test).

Regarding cell death, it was observed that cells that were positive for propidium iodide (PI), a marker of necrotic cell death (Figure 2A,B), increased from 0.15 ± 0.02% in control cells to 2.9 ± 0.8% in C18:3 cells. This parameter further increased from 3.0 ± 0.9% in ETOH cells to 38.6 ± 3.6% in the ETOH+C18:3 cells. Overall, these results suggest that the simultaneous presence of PUFA plus ethanol enhances the arrest of yeast growth via a robust induction of necrotic cell death.

### 3.2. Influence of PUFA on Lipid Peroxidation Levels in Yeast Treated with Ethanol

**Figure 3:**
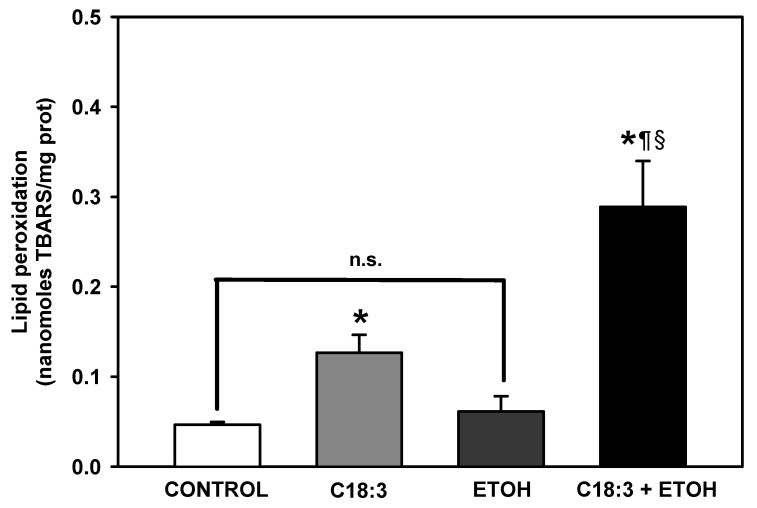
Effects of ethanol and C18:3 on lipid peroxidation in yeast cells. The results are expressed as nanomoles of thiobarbituric acid reactive substances (TBARS) per milligram of cells. The data are presented as the mean ± s.e. of *n* = 4. * *p* < 0.05 vs. CTRL; ^¶^ *p* < 0.05 vs. C18:3; ^§^ *p* < 0.05 vs. ETOH; n.s. non-statistical significance (one-way ANOVA with Tukey’s post-hoc test).

Lipid peroxidation was assessed to determine if necrotic cell death in ETOH + C18:3 cells was related to enhanced oxidative damage to membrane lipids. Lipid peroxidation levels (Figure 3) were 2.8-fold higher in C18:3 cells than in the control cells. Exacerbated lipid peroxidation was observed in ETOH+C18:3 cells, as lipid peroxidation was 6.8-fold higher in these cells than in control cells. In contrast, no changes in lipid peroxidation were observed in the cells with ETOH. This suggests that the presence of C18:3 exacerbates membrane damage by ethanol, which may be related to the enhanced necrotic cell death phenotype observed in ETOH+C18:3 cells (Figure 2).

### 3.3. Effects of Ethanol and PUFA on Cardiolipin Levels

**Figure 4:**
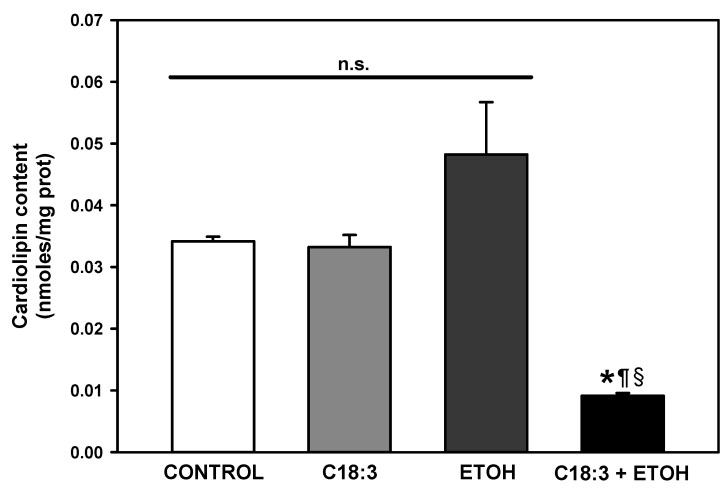
Effects of ethanol and C18:3 on cardiolipin levels in yeast mitochondria. The data are presented as the mean ± s.e. of *n* = 4. * *p* < 0.05 vs. CTRL; ^¶^ *p* < 0.05 vs. C18:3; ^§^ *p* < 0.05 vs. ETOH; n.s. non-statistical significance (one-way ANOVA with Tukey’s post-hoc test).

Cardiolipin levels were evaluated in isolated mitochondria to determine if exacerbated lipid peroxidation in ETOH+C18:3 cells induces diminution in cardiolipin content. In comparison to the control cells, the presence of PUFA did not modify cardiolipin levels in mitochondria from C18:3 cells (Figure 4) per se. Ethanol tended to increase cardiolipin levels in ETOH cells in a non-statistically significant fashion. On the contrary, ETOH + C18:3 cells exhibited a 73% decrease in cardiolipins levels with respect to control cells. This suggests that enhanced lipid peroxidation in ETOH + C18:3 cells involves damage to mitochondrial membranes and subsequent cardiolipin loss.

### 3.4. Influence of PUFA over the Effects of Ethanol on In Situ Mitochondrial Respiration and ROS Production

**Figure 5:**
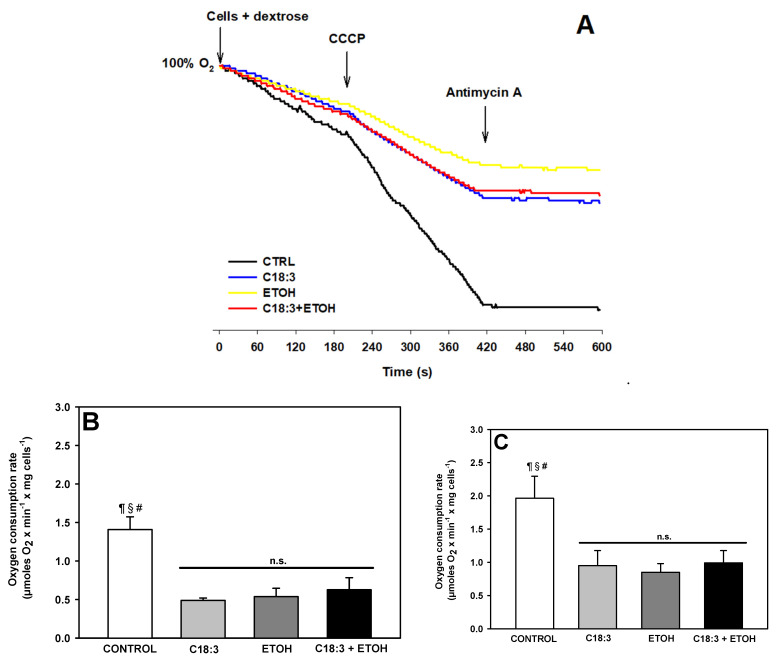
Effects of ethanol and C18:3 on in situ mitochondrial respiration in yeast. (**A**) Representative trace of respiration. Dextrose was added as substrate to stimulate state 4 respiration. The uncoupler, CCCP, was added to stimulate uncoupled respiration (state U). Antimycin was added to discriminate between non-mitochondrial and mitochondrial respiration. Quantification of respiration in states 4 (**B**) and U (**C**). The data are presented as the mean ± s.e. of *n* ≥ 4. ^¶^ *p* < 0.05 vs. C18:3; ^§^ *p* < 0.05 vs. ETOH; ^#^ *p* < 0.05 vs. C18:3 + ETOH; n.s. non-statistical significance (one-way ANOVA with Tukey’s post-hoc test).

**Figure 6:**
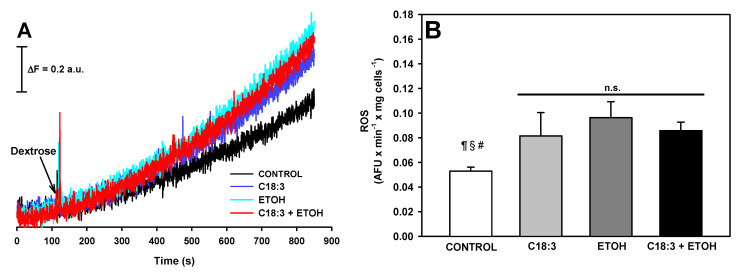
Effects of ethanol and C18:3 on reactive oxygen species (ROS) levels in yeast. (**A**) Representative trace of ROS. Cells were incubated with the fluorescent ROS probe H_2_DCFDA as indicated in Materials and Methods. Basal fluorescence was recorded and, where indicated, dextrose was added as substrate to stimulate ROS production. (**B**) Quantification of ROS production stimulated with dextrose. The results are expressed as arbitrary fluorescence units (AFU) per minute per milligram of cells. The data are presented as the mean ± s.e. of *n* ≥ 4. ^¶^ *p* < 0.05 vs. C18:3; ^§^ *p* < 0.05 vs. ETOH; ^#^ *p* < 0.05 vs. C18:3 + ETOH; n.s. non-statistical significance (one-way ANOVA with Tukey’s post-hoc test).

Antimycin A-sensitive respiration in whole yeast cells was assessed to determine if in situ mitochondrial function is impaired to a higher degree with ETOH + C18:3 than with only ETOH or C18:3. As observed in the representative traces of (Figure 5A), idling respiration (i.e., state 4) stimulated with dextrose as a substrate, and uncoupled respiration (i.e., in state U) stimulated with the uncoupler CCCP, decreased to a similar degree with any treatment in comparison to the control. The quantification of respiration data revealed a ~47% decrease in state 4 respiration (Figure 5B) and 52% in state U respiration (Figure 5C). In concordance with this result, representative traces of ROS production revealed an increase in this parameter at the same magnitude with all the treatments in comparison to the control. The quantification of these data revealed an increase in ~66% of all the treatments vs. the control group (Figure 6). Overall, these results suggest that mitochondrial dysfunction and increased ROS generation were common events involved in the deleterious effects of ETOH, C18.3, or ETOH + C18:3. Therefore, exacerbated lipid peroxidation and cardiolipin loss accompanied by mitochondrial dysfunction and ROS production is more important for enhanced necrotic cell death by ETOH + PUFA than only mitochondrial dysfunction or ROS production.

## 4. Discussion

The results of the present study show that PUFA plus ethanol severely impaired cell growth (Figure 1) and promotes a necrotic phenotype in the ETOH + C18:3 cells (Figure 2). This was associated with a striking increase in lipid peroxidation (Figure 3) and loss of cardiolipin (Figure 4). In contrast, ethanol in the absence of PUFA (i.e., in the ETOH cells) had no effect on lipid peroxidation levels (Figure 3), had a moderate effect on yeast growth that was similar to that observed in C18:3 cells, tended to increase cardiolipin levels (Figure 4), and slightly induced necrotic cell death like in C18:3 cells (Figure 2). The high levels of lipid peroxidation in the ETOH + C18:3 cells may be related to the ability of ethanol to increase the labile iron pool by disrupting mitochondrial iron homeostasis [11]. This may disrupt overall iron homeostasis in the cell, thereby causing increased levels of free iron in the cytosol [22]. As a consequence, augmented free iron levels could catalyze the generation of the highly oxidant hydroxyl radical (OH^•^) [13], leading to the enhancement of lipid peroxidation as OH^•^ initiates free radical attack in PUFA [23]. In turn, lipid peroxidation increases free iron levels in the mitochondria of yeast grown with PUFA [24]. This may be perpetuating the lipid peroxidation process by establishing a positive feed-forward loop of lipid peroxidation–iron release–lipid peroxidation, thereby explaining the higher levels of lipid peroxidation in ETOH + C18:3 cells (Figure 3). Conversely, as yeast without C18:3 does not contain PUFA in its membranes, there is no site for the attack of OH^•^ radicals in the acyl chains of phospholipids, thereby limiting lipid peroxidation and oxidative stress to other molecules than lipids.

The notable loss of cardiolipin in ETOH + C18:3 cells may be related to the fact that yeast readily incorporates PUFA into cardiolipin acyl chains [13]. Thus, the enrichment of cardiolipin with C18:3 may aggravate lipid peroxidation by ethanol, causing the eventual loss of cardiolipin molecules due to the formation of small aldehydes as final products of lipid peroxidation [3]. There was no decrease in cardiolipin levels in the C18:3 cells (Figure 4) and lipid peroxidation was about a third part of that observed in the ETOH + C18:3 cells. Yeast possesses the cardiolipin phospholipase, Cld1, which remodels peroxidized cardiolipin by substituting peroxidized acyl chains with linoleate [13]. Therefore, the lower levels of lipid peroxidation together with Cld1 activity may be limiting cardiolipin loss in C18:3 cells. On the contrary, excessive lipid peroxidation in the ETOH + C18:3 cells may be overcoming the ability of Cld1 to remodel cardiolipin, thereby augmenting cardiolipin loss in this group.

Respiration was inhibited equally in C18:3, ETOH, and ETOH+C18:3 cells in both state 4 (Figure 5B) and state U (Figure 5C). A full suppression of respiration in the ETOH + C18:3 cells due to the severe loss of cardiolipin may be expected, as observed in Figure 4. However, as demonstrated previously [25,26], the lack of cardiolipin does not fully impair the rate of respiration in yeast at moderate or high rates of oxidative phosphorylation and under optimal experimental conditions. Thus, the decreased rate of respiration in ETOH + C18:3 cells should be attributed to another factor other than cardiolipin loss, as the respiration rate in these cells was similar to that observed in both C18:3 and ETOH cells—despite the latter two having normal levels of cardiolipin when compared to control cells (Figure 4). The decrease in respiration in ETOH cells agrees with a previous report that 10% ethanol decreases respiration in state 4 and state U, which may be the result of oxidative damage in the mitochondria due to increased levels of free iron [11]. On the other hand, decreased respiration in the C18:3 cells may be associated with a moderate induction of lipid peroxidation (Figure 3), since lipid peroxidation is a key factor impairing the function of the ETC [27]. Thus, these factors may also be contributing to decreased respiration in ETOH+C18:3 cells. In agreement with the inhibition of respiration of a similar magnitude between the ETOH, C18:3, and ETOH + C18.3 cells, there was a parallel increase in ROS production of equal magnitude in all these cells (Figure 6). Therefore, the differences in necrosis, lipid peroxidation, and cardiolipin loss between the ETOH + C18:3 cells and the ETOH and C18:3 cells cannot be attributed to differences in ROS production.

A low percentage of cells underwent cell death, except for the ETOH + C18:3 cells (Figure 2). A high percentage of these cells internalized PI, indicating that ETOH + C18:3 cells die by necrosis. Plasma membrane disruption is a key step in necrotic cell death in both yeast [28] and animals [29]. Lipid peroxidation is a chief factor driving the disruption of both the plasma membrane and organelles in necrosis [29]. Moreover, the oxidation of cardiolipin and diminution in its content in the mitochondria of necrotic cells has been observed [30]. On this basis, it can be hypothesized that exacerbated lipid peroxidation induced by ethanol in ETOH + C18:3 cells (Figure 3) may lead to plasma membrane disruption and necrosis induction, as evidenced by the high percentage of ETOH + C18:3 cells positive to PI (Figure 4). Moreover, the drastic loss of cardiolipin of these cells may be contributing to the necrotic phenotype by decreasing stress resistance, as it has been demonstrated that cardiolipin depletion in yeast decreases mitochondrial fitness in stressful conditions like hyperosmolarity or high temperatures [25,26]. In agreement with this hypothesis, the ETOH cells exhibited negligible rates of necrosis (Figure 2), which may be due to the lipid peroxidation (Figure 3) and cardiolipin levels (Figure 4) remaining unchanged in comparison to the control cells.

*S. cerevisiae* is used in many industrial and biotechnological processes involving large scale fermentations, which implies the accumulation of ethanol [10]. Indeed, *S. cerevisiae* has been engineered to produce PUFA, which has been challenging due to some fatty acids negatively modifying stress tolerance in this yeast [31]. Hence, the study of the effects of PUFA on the tolerance to stress by ethanol is not trivial as it may give valuable information for the engineering of yeast for PUFA production. Taking into consideration the results of this study, the protection of yeast against lipid peroxidation and cardiolipin damage may be a useful strategy to improve yeast fitness during processes involving the accumulation of both ethanol and PUFA. Moreover, our results also highlight that targeting ROS production would not be enough to improve yeast resistance against necrotic cell death by ethanol plus PUFA, since ROS levels were not higher in the ETOH+C18:3 cells than in the C18:3 or ETOH cells (Figure 6). Finally, from a physiological point of view, the absence of PUFA in yeast membranes might be a mechanism for supporting high ethanol concentrations by preventing excessive membrane disruption by lipid peroxidation (Figure 3). This would lead to lower cardiolipin loss (Figure 4), lower necrosis induction (Figure 2), and a higher survival rate (Figure 1), despite the decreased mitochondrial function (Figure 5) and increased ROS production (Figure 6) observed in the ETOH cells.

## Data Availability

The raw data supporting the conclusions of this article will be made available by the corresponding author, without undue reservation.

## Supplementary Materials

The following supporting information can be downloaded at: https://www.mdpi.com/article/10.3390/life12071052/s1, Figure S1: Positive controls of necrotic cell death. Cells were grown for 24 h under control conditions or with 10% ethanol plus linolenic acid (ETOH + C18:3). Cells were harvested and incubated for 90 min at 30 °C (control) or 50 °C (heat shock). Cells positive for propidium iodide (PI) were assessed by flow cytometry as indicated in materials and methods. Signals in the V2-L region correspond to live cells; signals in the V2-R region correspond to necrotic, PI—positive cells; Figure S2: Representative time—trace of mitochondrial respiration for assessing quality control of the procedure for mitochondria isolation. 0.5 mg/mL mitochondrial protein was resuspended in 2.5 mL of a buffer with 10 mM MES-TEA (pH 6.0). After exhaustion of 10 mM ADP, respiration back to state 4 (i.e., idling respiration) and 100 s later, 20 mM ADP was added to induce state 3 respiration (i.e., phosphorylating respiration). 5 µg Antimycin A was added before ADP exhaustion to discriminate non-mitochondrial oxygen consumption; Figure S3: Positive control of cardiolipin detection with 10-N-nonyl acridine orange. Cardiolipin liposomes were prepared by sonicating a mixture of 1.7 mg cardiolipin (Sigma-Aldrich, St. Louis, MO, USA) with 1 mL 25 mM KH_2_PO_4_. Six cycles of sonication were applied at 40 W for 2.5 min each under a N_2_ stream. Liposomes were placed in 1 mL 0.6 M mannitol/10 mM Tris (pH 7.4) at concentrations of 0.03, 0.08, 0.17, 0.27, 0.34 and 0.54 mg/mL. These solutions were mixed with 150 µM\10-N-nonyl acridine orange and incubated for 15 min in ice. Then, these mixtures were centrifuged at 15000 rpm for 5 min. The absorbance of the supernatant was measured at 495 nm.
