# Supplementary material for: Linolenic Acid Plus Ethanol Exacerbates Cell Death in Saccharomyces cerevisiae by Promoting Lipid Peroxidation, Cardiolipin Loss, and Necrosis
Source: Life (Basel). 2022 Jul 14;12(7):1052. doi: 10.3390/life12071052 (PMC9320082; doi:10.3390/life12071052)

Supplementary Files

Figure S1: Positive controls of necrotic cell death. Cells were grown for 24 h under control conditions or with 10% ethanol plus linolenic acid (ETOH+C18:3). Cells were harvested and incubated for 90 min at 30 °C (control) or 50 °C (heat shock). Cells positive for propidium iodide (PI) were assessed by flow cytometry as indicated in materials and methods. Signals in the V2-L region correspond to live cells; signals in the V2-R region correspond to necrotic, PI – positive cells.

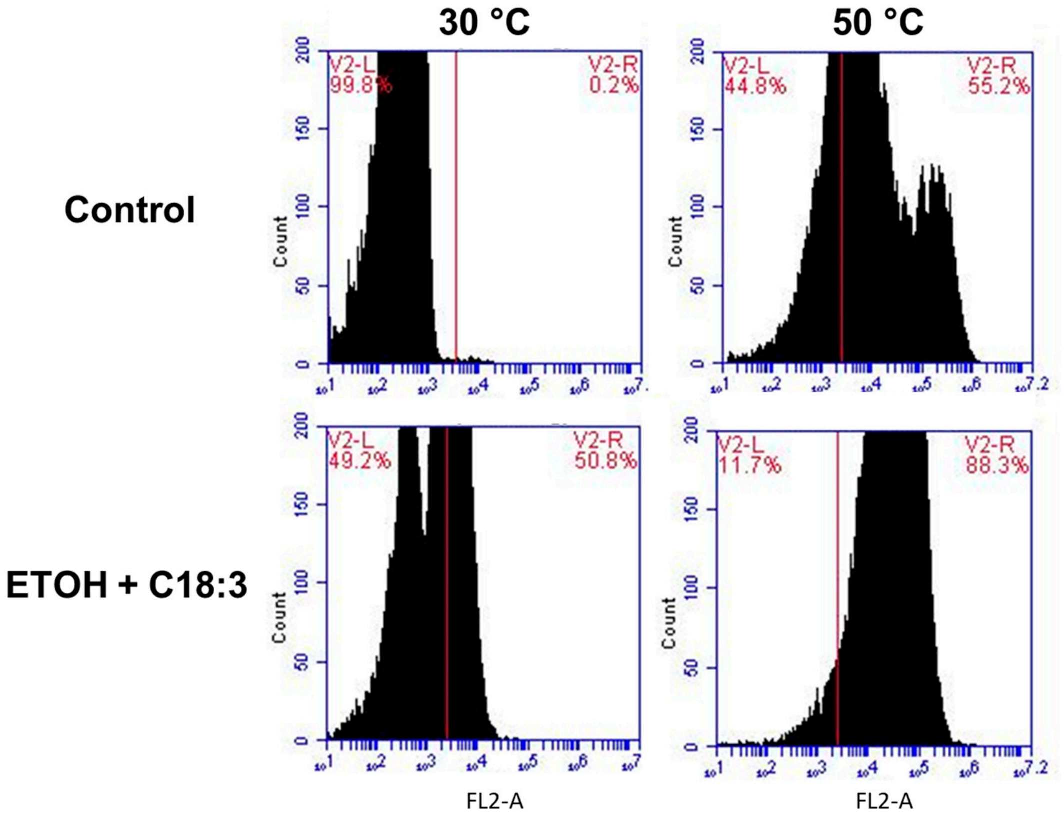

Figure S2: Representative time - trace of mitochondrial respiration for assessing quality control of the procedure for mitochondria isolation. 0.5 mg/mL mitochondrial protein was resuspended in 2.5 mL of a buffer with 10 mM MES-TEA (pH 6.0). After exhaustion of 10 mM ADP, respiration back to state 4 (i.e., idling respiration) and 100 s later, 20 mM ADP was added to induce state 3 respiration (i.e., phosphorylating respiration). 5 µg Antimycin A was added before ADP exhaustion to discriminate non-mitochondrial oxygen consumption.

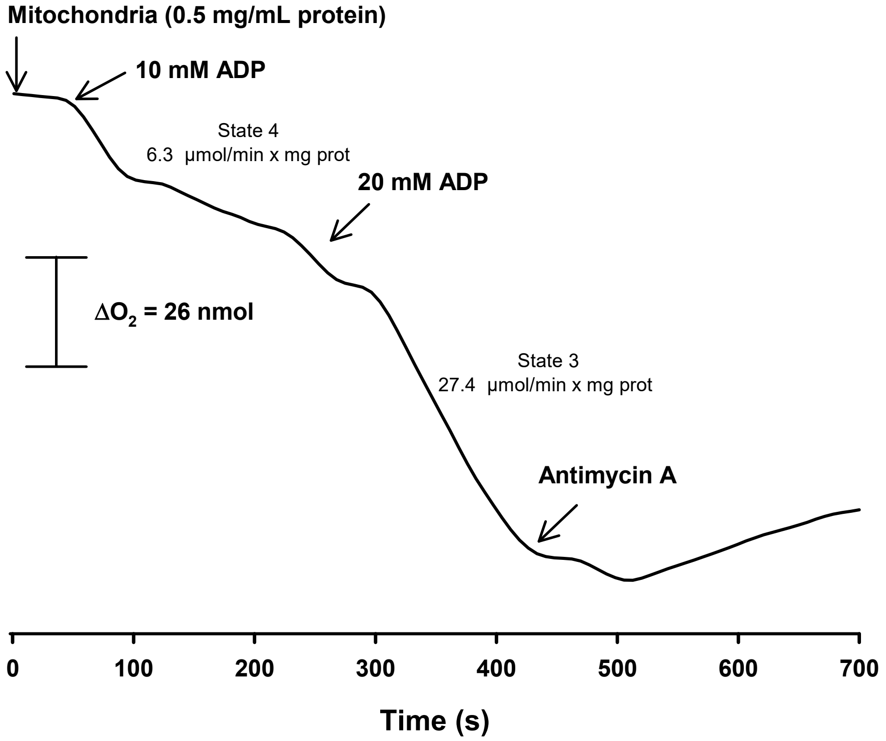

Figure S3. Positive control of cardiolipin detection with 10-N-nonyl acridine orange. Cardiolipin liposomes were prepared by sonicating a mixture of 1.7 mg cardiolipin (Sigma-Aldrich, St. Louis, MO, USA) with 1 mL 25 mM KH_2PO_4. Six cycles of sonication were applied at 40 W for 2.5 min each under a N_2 stream. Liposomes were placed in 1 mL 0.6 M mannitol/10 mM Tris (pH 7.4) at concentrations of 0.03, 0.08, 0.17, 0.27, 0.34 and 0.54 mg/mL. These solutions were mixed with 150 μM 10-N-nonyl acridine orange and incubated for 15 min in ice. Then, these mixtures were centrifuged at 15000 rpm for 5 min. The absorbance of the supernatant was measured at 495 nm.

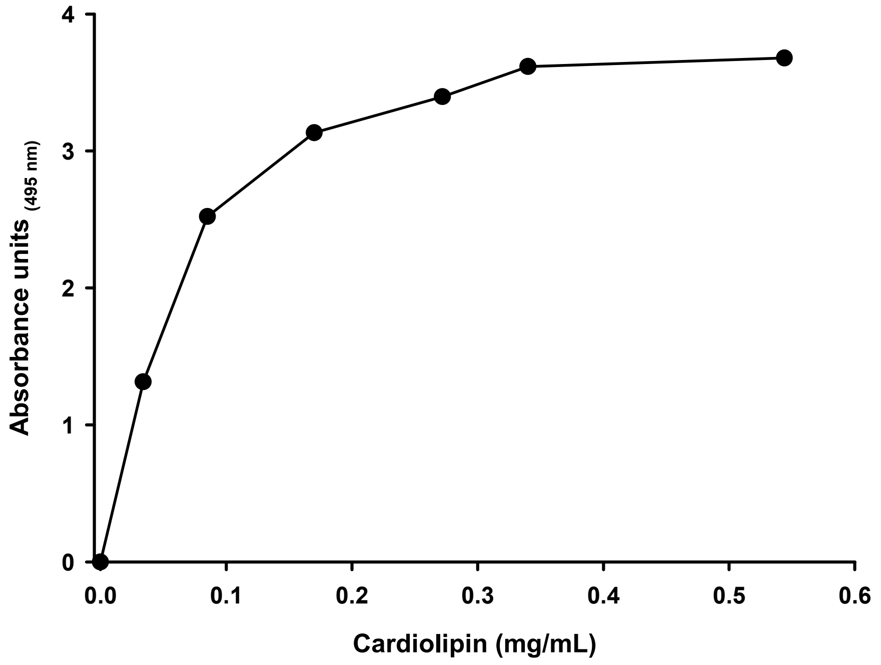

Supplement: Supplementary file 1 [file life-12-01052-s001.zip › life-1818609-supplementary.pdf]
